# The CDK9/Cyclin T1 subunits of P-TEFb in mouse oocytes and preimplantation embryos: A possible role in embryonic genome activation

**DOI:** 10.1186/1471-213X-11-33

**Published:** 2011-06-03

**Authors:** Reza K Oqani, Hong R Kim, Yun F Diao, Chang S Park, Dong I Jin

**Affiliations:** 1 Department of Animal Science & Biotechnology, Research Center for Transgenic Cloned Pigs, Chungnam National University, Daejeon, Korea

## Abstract

**Background:**

Two stages of genome activation have been identified in the mouse embryo. Specifically, minor transcriptional activation is evident at the one-cell stage and a second major episode of activation occurs at the two-cell stage. Nuclear translocation of RNA polymerase II and phosphorylation of the C-terminal domain (CTD) of the largest enzyme subunit are major determinants of embryonic genome activation. P-TEFb, the Pol II CTD kinase, regulates transcriptional elongation via phosphorylation of the serine 2 residues of the CTD.

**Results:**

Here, we show that the CDK9 and cyclin T1 subunits of P-TEFb are present in mouse oocytes and preimplantation embryos. Both proteins translocate to pronuclei at the late one-cell stage and are predominantly localized in nuclei at the two-cell stage. We additionally examine the effects of the CDK9-specific inhibitor, flavopiridol, on mouse preimplantation development. Our data show that treatment with the drug results in mislocalization of CDK9, cyclin T1, and phosphorylated Pol II, as well as developmental arrest at the two-cell stage.

**Conclusions:**

A change in CDK9 localization from the cytoplasm to the pronucleus occurs at the time of minor embryonic genome activation, and CDK9 accumulation at the two-cell stage is evident, concomitant with major transcriptional activation of the embryonic genome. Moreover, CDK9 inhibition triggers a developmental block at the two-cell stage. Our findings clearly indicate that CDK9 is essential for embryonic genome activation in the mouse.

## Background

The maternal-zygotic transition is a critical event in early mouse embryogenesis. This transition transforms the highly differentiated oocyte into a totipotent blastomere, and is complete by the two-cell stage. During this transition, maternal mRNAs are degraded and the embryonic genome is activated [1]. Genome activation results in the replacement of transcripts common to both the oocyte and the embryo and the generation of new transcripts necessary for further development. Development of mouse embryos unable to accomplish genome activation is terminated at the two-cell stage.

In the mouse, two transcriptional stages have been identified: a minor transcriptional wave at the one-cell stage, and a second major wave at the two-cell stage [2]. These findings are supported by the results of experiments showing that the one-cell stage features significant RNA polymerase II (Pol II)-dependent incorporation of bromouridine triphosphate (BrUTP) into RNA, and RNA synthesis is accompanied by an obvious increase in BrUTP incorporation at the two-cell stage. BrUTP uptake during the one-cell stage is only 40% of that at the two-cell stage. The higher levels of BrUTP incorporation seen at the two-cell stage are maintained at subsequent developmental stages [2-4].

In eukaryotes, Pol II is responsible for transcription of mRNA and most small nuclear RNAs. Transcription of class II genes requires the coordinated assembly of Pol II and six general transcription factors; these are TFIIA, TFIIB, TFIID, TFIIE, TFIIF, and TFIIH [5]. Transcriptional initiation commences with formation of the first phosphodiester bond and phosphorylation of serine 5 (Ser5) (by TFIIH) in the C-terminal domain (CTD) of the largest subunit of Pol II. The CTD of Pol II, composed of a highly conserved tandemly repeated heptapeptide motif (YSPTSPS), undergoes extensive phosphorylation and dephosphorylation during the transcription cycle. Pol II exists primarily in two major forms; specifically, with an unphosphorylated CTD (UnP CTD) and with an extensively phosphorylated (mainly at Ser2 and/or Ser5) CTD, designated the hyperphosphorylated form. The CTD of Pol II is a major target of CDK9 kinase activity, and the distinct phosphorylation states of the enzyme are associated with different functionalities. This oscillation of CTD phosphorylation regulates recruitment of various factors required throughout transcription [6].

Positive transcription elongation factor b (P-TEFb), also termed CDK9/cyclin T1, the metazoan Pol II CTD kinase, regulates transcription elongation by phosphorylating Ser2 of the CTD and Negative Elongation Factor-E (NELF-E) [7-10]. Phosphorylation of NELF-E removes the block against early transcriptional elongation induced by binding of the NELF complex to the nascent transcript [11,12]. Within the cell, P-TEFb exists in two forms, designated the large and the free forms [13,14]. The kinase-active free form contains CDK9 and one of several cyclin regulatory subunits (cyclin T1, cyclin T2a, cyclin T2b, or cyclin K), with cyclin T1 being predominant in many cell types [15,16]. The kinase-inactive large form of P-TEFb additionally contains 7SK RNA [13,14] and either hexamethylene bisacetamide-induced protein 1 (HEXIM1) [17,18] or HEXIM2 [19]. In HeLa cells, 50-90% of P-TEFb exists as the large form, with the remaining protein being in the kinase-active free form [13,14,18,19]. It is hypothesized that the large form of P-TEFb serves as a reservoir for the free form.

Phosphorylation of the CTD plays a further important role in co-transcriptional mRNA processing *in vivo*. Specifically, the phosphorylated protein serves as a binding platform for factors involved in 5' end capping, splicing, and 3' end-processing of pre-mRNA, as well as chromatin modification [20].

P-TEFb is required for transcription of most genes, including heat-shock genes and *c-Myc*, and also for HIV-1 transcription by TAT [21]. Shim *et al. *(2002) reported that P-TEFb was, in general, essential for expression of early embryonic genes in *Caenorhabditis elegans *[22]. Additionally, Ser2 phosphorylation is eliminated upon genetic inactivation of CDK9 or its cyclin T1 subunit. *C. elegans *development is arrested at the 100-cell stage in the absence of cyclin T1 or CDK9; this is precisely what is noted upon knockdown of the large subunit of Pol II. Experiments using yeast and *Drosophila *have shown that CDK9 is vital for all of appropriate 3' end-processing of pre-mRNA [23,24], gene expression, histone methylation, and elongation factor recruitment [25].

Flavopiridol is a potent anti-cancer and -HIV therapeutic agent currently under investigation in clinical trials [26,27]. This compound is the most potent inhibitor of P-TEFb identified to date and the first reported CDK inhibitor that acts in a manner that is not competitive with ATP [28]. Flavopiridol inhibits transcriptional elongation *in vitro *by targeting CDK9; the IC_50 _value of this effect is 5-10-fold lower than that noted when effects on other CDKs are assessed [29]. Nuclear run-on transcription assays have shown that flavopiridol inactivates P-TEFb and blocks most Pol II-mediated transcription *in vivo *[29].

To the best of our knowledge, the expression patterns and subcellular localization of CDK9 and cyclin T1 in mammalian oocytes and preimplantation embryos have not been investigated. Herein, we show, for the first time, that both CDK9 and cyclin T1 are present in pre-ovulatory mouse oocytes, through to the blastocyst stage. We further explore the effects of a CDK9-specific inhibitor, flavopiridol, on mouse embryo preimplantation development and CDK9 localization in early embryos.

## Results

### CDK9 and cyclin T1 in oocytes and embryos

**Figure 1:**
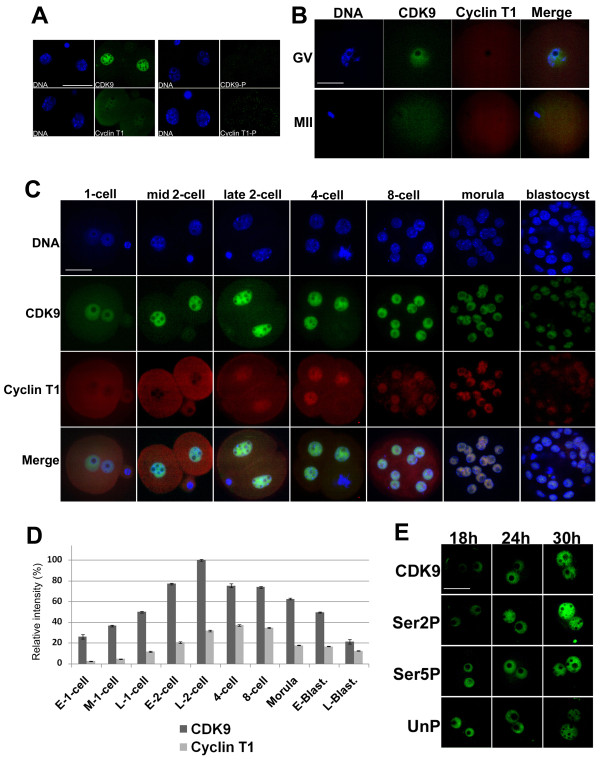
**Expression and subcellular localization of CDK9 and cyclin T1 in mouse oocytes and preimplantation embryos**. **A**. The antibodies against CDK9 and cyclin T1 used in the present study specifically recognize the corresponding antigens in mouse embryos. Late two-cell embryos were separately immunostained with working dilutions (1:50) of either anti-CDK9 or -cyclin T1 antibodies (left) or with such antibodies pre-incubated with 10-fold molar excesses of the peptide antigens (right). Bar: 50 μm. **B**. Expression of CDK9 and cyclin T1 in mouse germinal vesicles and MII oocytes. Note that, in both instances, no fluorescent signals emanated from the nucleolar area. Bar: 50 μm. **C**. Time course of CDK9 and cyclin T1 expression at defined preimplantation stages by immunocytochemistry. Rabbit polyclonal and mouse monoclonal antibodies were used for immunolocalization of CDK9 (green) and cyclin T1 (red), respectively. Chromatin was counterstained with DAPI. Bar: 50 μm. **D**. Relative intensities of fluorescent signals from both anti-CDK9 and -cyclin T1 antibodies in postfertilization embryos. Samples from all stages were simultaneously processed for immunostaining and images were taken at the same laser power, thus enabling direct comparison of signal intensities. The results are mean values from at least five embryos. The fluorescence intensity at late 2-cell stage has been set as 100%. **E**. Nuclear translocation of CDK9 and Pol II CTD phosphoisoforms after fertilization. One-cell embryos were immunostained with antibodies against CDK9, Ser2P-, Ser5P-, and UnP-Pol II CTD at 18 h, 24 h, and 30 h after hCG injection (h phCG). Bar: 50 μm.

To determine if antibodies against CDK9 or cyclin T1 specifically recognized the corresponding antigens in mouse embryos, we conducted a binding competition assay in which the antigen peptides were separately incubated with the appropriate antibodies prior to immunofluorescence staining. Two-cell embryos were stained with antibody against either CDK9 or cyclin T1 alone, or the antibody-peptide mixture, and compared. Figure 1A shows that CDK9 or cyclin T1 peptide completely blocked the binding of the corresponding antibody and abolished the signal therefrom, thus indicating that each antibody was specific. Next, CDK9 and cyclin T1 levels were quantitated in mouse embryos. Both CDK9 and cyclin T1 were maternally present in mouse pre-ovulatory oocytes (Figure 1B). Signal from antibody recognizing the CDK9 protein was detected in all growing and fully grown germinal vesicle oocytes including both NSN and SN configurations, and was predominantly nuclear in location. However, only a faint nucleo-cytoplasmic signal from antibody against cyclin T1 was evident in these oocytes. Both CDK9 and cyclin T1 were weakly detected in the cytoplasm of mature oocytes. After fertilization, both proteins were present at all stages of preimplantation development (Figure 1C). Shortly after fertilization, cyclin T1 remained in the cytoplasm but increased in both pronuclei at the late one-cell stage whereas CDK9 translocated to nuclei at the mid one-cell stage. In the majority of instances (24 of 28 zygotes analyzed), signal from CDK9 protein was more intense in the male pronucleus. The strongest signal from CDK9 was observed in late two-cell-stage embryonic nuclei (Figure 1D). Cyclin T1 became predominantly localized to the nucleus only at the late two-cell stage. Subsequently, nuclear distribution of cyclin T1 was detected at all stages. After blastocyst formation, signals from CDK9 and cyclin T1 fell rapidly in intensity. In all stages examined, both CDK9 and cyclin T1 were dissociated from mitotic chromosomes (see discussion). Figure 1C showed a representative mitotic status of one nucleus in 4-cell embryo.

### Subcellular localization of Pol II phosphoisoforms in the fertilized embryo

CTD phosphorylation status and subcellular localization were examined at the time of CDK9 nuclear accumulation in fertilized eggs, using monoclonal antibodies recognizing hypo-, Ser2-, and Ser5-phosphorylated Pol II CTD. The monoclonal antibody H14 recognizes phosphorylated Ser5 residues within the heptapeptide repeats of the CTD, generated via TFIIH activity and required for transcription initiation. The transition from initiating to elongating Pol II complexes occurs when CDK9 phosphorylates Ser2 residues within the CTD heptads, and the monoclonal antibody H5 recognizes these phosphorylated epitopes. All three phosphoforms of the CTD were present in both paternal and maternal pronuclei shortly after pronuclear formation (Figure 1E). The nuclear concentration of both Ser2P and Ser5P increased following pronuclear formation, and this rise continued during the course of the first cell cycle. In addition, the concentrations of both phosphoforms were greater in the male pronucleus in all zygotes examined. Correspondingly, a fall in the level of pronuclear hypophosphorylated CTD was evident following pronucleus formation.

### Effects of flavopiridol on embryo development

**Figure 2:**
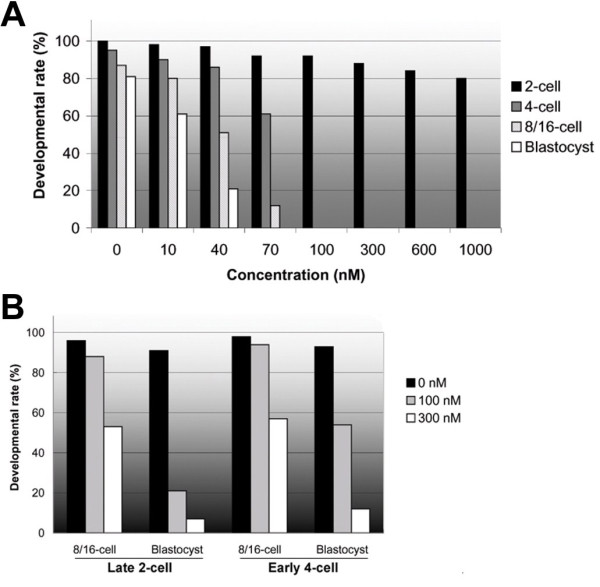
**Effects of flavopiridol on mouse embryo development *in vitro***. **A**. One-cell embryos collected at 20 h phCG were cultured in KSOM medium containing 0, 10, 40, 70, 100, 300, 600, or 1,000 nM flavopiridol, for 84 h. **B**. Late two-cell- or early four-cell-stage embryos were recovered at 50 h phCG and cultured in KSOM medium without or with 100 nM or 300 nM flavopiridol, for 60 h.

To ascertain whether CDK9 was involved in embryo development, we examined the influence of a specific inhibitor, flavopiridol, on *in vitro *development of mouse embryos by addition of the compound to culture medium at one-cell (18 hphCG) and late two-cell or early four-cell (50 hphCG) stages. To establish the minimum effective drug concentration, we compared the effects of various doses of flavopiridol on embryo development. One-cell embryos were cultured in the presence of increasing concentrations (0, 10, 40, 70, 100, 300, 600, and 1000 nM) of flavopiridol. In the absence of the drug, all pronuclear zygotes developed to the two-cell stage after 24 h of culture and 81% of embryos reached the blastocyst stage after 80 h (Figure 2A). Development to the two-cell stage was slightly reduced upon addition of increasing concentrations of flavopiridol. With 70 nM drug, only a small proportion (12%) of embryos reached the 8/16-cell stage and none developed beyond this step. In the presence of 100 nM flavopiridol, the majority of embryos stopped developing at the two-cell stage and none reached the four-cell stage. At the higher concentrations examined, at least 80% of zygotes completed first mitosis. Accordingly, we concluded that 100 nM was the minimum effective concentration of flavopiridol inhibiting CDK9 activity in the embryo. Next, late two-cell/early four-cell embryos were treated with this concentration of flavopiridol to determine the effects of CDK9 inhibition on embryo development beyond the two-cell stage. In the presence of 100 nM flavopiridol, most (88%) late 2-cell embryos developed to the 8/16-cell stage and 20% reached to blastocyst (Figure 2B). At the same concentration, 92% of early 4-cell embryos divided to 8/16-cell embryos and 56% formed blastocysts. At 3-fold increased concentration of flavopiridol, 51% of late-2-cell embryos developed to 8/16-cell stage and 8% formed blastocysts. At this concentration, 58% of early 4-cell embryos divided to 8/16 cell and 10% formed blastocysts. These suggest that the developmental arrest observed at the two-cell stage may not attributable to a cytotoxic action of flavopiridol or to other drug effects, such as inhibition of other CDKs, including CDK1 or CDK4. Our results clearly indicated that treatment with a CDK9 inhibitor from the early one-cell stage caused embryos to arrest at the two-cell stage, suggesting the involvement of CDK9 in transition of embryos from the two- to the four-cell stage.

### Effects of flavopiridol on localization of CDK9/cyclin T1 and Pol II

**Figure 3:**
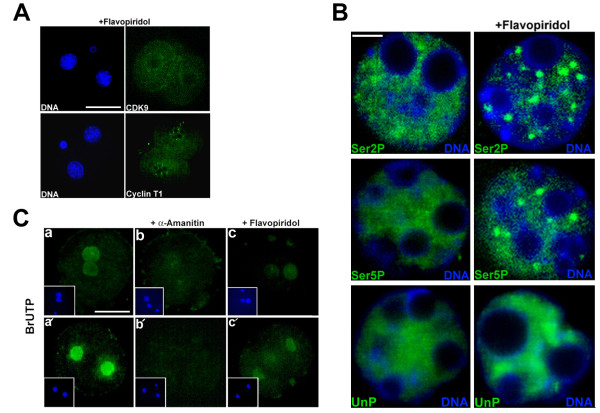
**Effects of flavopiridol on localization of CDK9/cyclin T1 and phosphorylated Pol II CTD, and on transcription in embryos**. **A**. Early one-cell stage mouse embryos were cultured in the presence of 100 nM flavopiridol to the two-cell stage and the embryos were next subjected to immunolabeling to detect cyclin T1 and CDK9 (separately). The experiment was repeated three times; 30 embryos were analyzed in total. Bar: 50 μm. **B**. Early one-cell stage mouse embryos were cultured in the absence (left) or presence (right) of 100 nM flavopiridol to the two-cell stage and immunostained with antibodies detecting phosphorylated serine 2 (Ser2P), phosphorylated serine 5 (Ser5P) or unphosphorylated (UnP) Pol II CTD. The Figure depicts CTD phosphoforms staining in one nucleus of two-cell embryos. The experiment was repeated three times. Bar: 5 μm. **C**. Early one-cell-stage embryos were cultured in KSOM for 9 h or 32 h in the absence (left) or presence of 250 μg/ml α-amanitin (middle) or 100 nM flavopiridol (right) and subjected to *in situ *run-on transcription assay. Embryos were permeabilized and incubated in transcription buffer containing 100 μM BrUTP. a, control late one-cell stage embryo. b, late one-cell stage embryo treated with α-amanitin. c, late one-cell stage embryo treated with flavopiridol. a', control two-cell stage embryo. b', two-cell stage embryo treated with α-amanitin. c', two-cell stage embryo treated with flavopiridol. The staining evident at the periphery is attributable to non-specific binding of the secondary antibody to the zona pellucida. Bar: 50 μm.

To clarify the effects of inhibition of CDK9 kinase activity on the subcellular status of CDK9 and cyclin T1, embryos were immunostained after 30 h of treatment with flavopiridol *in vitro*. Impairment of nuclear localization of both proteins following drug treatment was clearly evident (Figure 3A). To determine the effect of inhibition of CDK9 by flavopiridol, we conducted immunofluorescence labeling of the Pol II CTD. Our immunostaining experiments revealed that, at the two-cell stage, flavopiridol-treated embryos showed obviously aberrant nuclear localization of all forms of Pol II CTD (Figure 3B). In control two-cell embryos, the Ser2P, Ser5P and UnP CTD were distributed uniformly throughout the nucleoplasm, excluding the nucleolar area. In contrast, in flavopiridol-treated two-cell embryos, Ser2P and Ser5P CTD were concentrated into uneven, dot-like structures. UnP CTD also showed a different distribution in flavopiridol-treated two-cell embryos compared with that of control embryos. It seems that UnP CTD was accumulated in some parts of nucleoplasm after treatment with the drug (Figure 3B).

### Effect of flavopiridol on embryo transcription

To directly measure of Pol II-dependent transcription in fertilized embryos in the presence of flavopiridol, nascent RNA chains were labeled in situ by incorporation of bromouridine 5'-triphosphate (BrUTP). One-cell embryos were cultured in the presence of either 250 μg/ml α-amanitin or 100 nM flavopiridol. This concentration of α-amanitin has been shown to prevent all RNA polymerase-dependent transcription in eukaryotic cells. Nuclear labeling was obvious in both male and female pronuclei of untreated embryos (Figure 3C, a). Confocal microscopy showed that fluorescence intensity clearly differed among control, α-amanitin-, and flavopiridol-treated embryos. Strong nuclear labeling was observed in control 2-cell embryos, whereas no nuclear signal was detected in 2-cell embryos treated with 250 μg/ml α-amanitin (Figure 3C, a' and 3b'). In flavopiridol-treated embryos, however, the fluorescence signal was dramatically decreased compared with that of untreated embryos; only a very faint nuclear signal was detectable (Figure 3C, c and 3c'). This is explained by the fact that Pol I and Pol III transcriptional activities may not be affected by the inhibition of CDK9.

## Discussion

In the present study, we demonstrate that CDK9 and its regulatory partner, cyclin T1, are present in mouse oocytes as well as during all preimplantation stages of development (Figure 1B,C). In immunofluorescence experiments, signals from both proteins decreased only during blastocyst expansion. In view of the finding that both proteins are present as early as the GV oocyte stage including NSN and SN oocytes, it appears that the relevant products are translated from maternal messages at this stage. CDK9 was predominantly nuclear, whereas cyclin T1 showed a nucleo-cytoplasmic distribution in immature oocytes. This observation is consistent with the finding that GV oocytes are transcriptionally active [30]. During oogenesis, an important transition takes place at the level of gene expression, as transcriptionally active (NSN) chromatin becomes silenced (SN) during meiosis. Accordingly, chromatin is modified and transcription factors are generally excluded from compact chromosomes. Some lines of evidence indicate that CDK9 is recruited to mitotic chromosomes at telophase in somatic cells [31,32], but our observations showed that CDK9 is not recruired to either meiotic or preimplantation mitotic chromosomes.

After fertilization, cyclin T1 was uniformly distributed throughout the cytoplasm and CDK9 was predominantly localized in the nucleus. At the mid one-cell stage, nucleoplasmic signals from CDK9 increased and concurrently, cyclin T1 accumulated within pronuclei. A priviuous study showed that Pol II subunits as well as other components of the basal transcription machinery are maternally contributed to the cytoplasm of the early embryo and translocate from the cytoplasm to the nuclei immediately prior to embryonic genome activation [33]. As the P-TEFb complex is a CTD kinase, nuclear accumulation of CDK9 and cyclin T1 at the late one-cell stage is consistent with nuclear translocation of the phosphorylated CTD during this period (Figure 1C and 1E), concomitant with transcriptional activation of the embryonic genome [34]. Thus, the change in CDK9 localization from the cytoplasm to pronuclei may be closely related to the minor phase of embryonic genome activation. In most instances, signals from both proteins were more intense within male pronuclei, in accordance with earlier activation of transcription in this pronucleus [34]. Although it is unclear whether the regulated translocation of the basal transcriptional machinery causes a transcriptional onset, or whether basal transcriptional components accumulate in early embryonic nuclei because of transcriptional activation, we hypothesize that nuclear translocation of factors such as CDK9, necessary for the transition from the initiation to elongation phase of transcription, regulates the onset of productive transcription in the embryo. In our experiments, the strongest signal from CDK9 was observed at the two-cell stage, during which cyclin T1 was predominantly localized in nuclei (Figure 1C). This is the time of major embryonic genome activation in the mouse. Thus, extensive nuclear accumulation of CDK9 and cyclin T1 at the two-cell stage appears to be significantly associated with the major step of embryonic genome activation.

Our *in situ *run-on transcription assay coupled with fluorescence microscopy showed that, compared with untreated embryos, BrUTP incorporation into nascent RNAs was reduced in flavopiridol-treated embryos, suggesting that transcription was defective in such embryos (Figure 3C). In the presence of a high concentration of α-amanitin (250 μM), sufficient to block the actions of all RNA polymerases, no BrUTP incorporation was evident. However, in the presence of flavopiridol, a faint nuclear signal was seen, likely due to Pol I- and Pol III-dependent transcription that was not inhibited by flavopiridol. In addition, treatment of embryos with flavopiridol resulted in aberrant localization of CDK9, cyclin T1, and phosphorylated Pol II CTD (Figure 3A and 3B). Our experiments showed that treatment with flavopiridol changed evenly distributed Ser2P and Ser5P CTD to uneven dot-like structures (speckles) in 2-cell embryo nuclei (Figure 3B). This is a common feature of Pol II and some other components of transcription machinery to form large and round speckles after inhibition of transcription [35]. This can be seen in our experiments. However, treatment with flavopiridol did not change unphosphorylated Pol II CTD (UnP) to dot-like structures in 2-cell embryos. Rather, it seems that UnP CTD is accumulated in some parts of nucleoplasm. This accumulation may be due to lack of Ser2 and/or Ser5 phosphorylation of the CTD in treated embryos. By increasing concentration of inhibitors like α-amanitin or DRB, the number of these speckles decreases and their sizes increase. And as seen in Figure 3A, CDK9 does not form speckles in treated embryos. That is obvious since in these embryos CDK9 fails to enter the nucleus. Complete absence of nuclear CDK9 in treated 2-cell embryos may be due to long exposure of them to CDK9 inhibitor. These data clearly imply that nuclear translocation of CDK9, followed by functional activity thereof, are essential for genomic activation in the mouse embryo. Previous studies have shown that cyclin T1 is predominantly a nuclear protein distributed throughout the nucleoplasm, and that protein signal intensity is elevated at discrete foci (termed nuclear speckles) [36]. CDK9 is present principally in the nucleus, but can additionally be visualized in the cytoplasm, and thus shuttles between the two cellular compartments [36]. A recent study found that nuclear localization of CDK9 requires that the kinase be catalytically active; the cited work showed that catalytically inactive kinase mutants failed to accumulate in the nucleus, rather remaining diffusely distributed in a subcellular localization [37].

It has been reported that the CDK9 C-terminal tail region contains Ser and Thr residues serving as the major phospho-acceptor sites for autophosphorylation. Mutation of these residues affects autophosphorylation but is not essential for binding of CDK9 to cyclin T1 or for phosphorylation of heterologous substrates [38,39]. Flavopiridol effectively blocks P-TEFb kinase activity [28,29], possibly explaining the aberrant localization of CDK9/cyclin T1 in the flavopiridol-treated two-cell embryos of our experiments. Although previous work showed that the long-term exposure of mammalian cells to high concentrations of flavopiridol resulted in G1-S arrest associated with loss of CDK2 and CDK4 [40], a very recent study revealed that CDK1 is the only essential cell cycle CDK [41]. In effect, mouse embryos lacking all interphase CDKs (CDKs 2, 3, 4, and 6) undergo organogenesis and develop to mid-gestation [41]. Only CDK1 inactivation results in failure to develop to the morula and blastocyst stages. In addition, our observations indicated that exposure of one-cell stage embryos to flavopiridol caused two-cell developmental block, but exposure of embryos to drug after embryonic genome activation did not result in cell cycle arrest (Figure 2). Together, the data suggest that the developmental arrest observed at the two-cell stage is caused by inactivation of CDK9 and may not attributable to the cytotoxicity of flavopiridol or to a drug effect on CDKs involved in regulation of the cell cycle. Our results thus indicate that CDK9 could play an important role in embryonic genome activation in the mouse.

## Conclusions

CDK9 and cyclin T1, subunits of the positive transcription elongation factor P-TEFb, are present and functional in mouse oocytes and early embryos. Inhibition of the kinase activity of P-TEFb using a CDK9-specific inhibitor, flavopiridol, caused defects in transcription, abnormal cellular P-TEFb localization, and developmental arrest at the time of genome activation in mouse two-cell stage embryos. Our results reveal that CDK9 function is essential for embryonic genome activation in the mouse.

## Methods

### Preparation of *in vivo*-derived mouse oocytes and embryos

All animal care and use procedures were approved by the Institutional Animal Care and Use Committee of Chungnam National University. Oocytes at the germinal vesicle stage were obtained from B6D2 F1 female mice (Charles River) as cumulus-oocyte complexes (COCs). Five- to seven-week old females were induced to superovulate by injection with 5 IU PMSG (Sigma) and sacrificed 48 h later. Ovaries were recovered in FHM medium (Millipore). COCs were mechanically removed and oocytes were washed by pipetting in FHM containing 0.1% (w/v) hyaluronidase (Sigma). Oocytes containing germinal vesicles were collected and subjected to immunofluorescence staining. Mature MII oocytes were collected as COCs after PMSG injection, followed by injection of 5 IU hCG (Sigma) after 44 h. Mice were killed 18 h after hCG (hphCG) injection. COCs were removed from oviducts into FHM and oocytes were denuded using hyaluronidase. Mature oocytes were washed in PBS-PVA and subjected to immunofluorescence staining. To obtain zygotes and embryos, female mice were coupled with males after hCG injection and killed 18, 24, 30, 42, 50, 64, 72, 88, 96 and 110 hphCG to recover early, mid, late 1-cell, early, late 2-cell, 4-cell, 8/16-cell embryos, morula, early and late blastocysts, respectively. Zygotes and embryos were washed in PBS-PVA and subjected to immunofluorescence staining.

### Culture and treatment of embryos *in vitro*

To determine the effects of flavopiridol on embryo development, early 1-cell or late 2-/early 4-cell stage embryos were recovered 18 or 50 h phCG, respectively, and cultured without or with flavopiridol. The required drug concentrations were prepared from stock solution diluted in KSOM (Millipore). Groups of 25-30 embryos were placed in warmed 40 μL droplets of culture medium, covered with mineral oil (Sigma), and cultured under 5% (v/v) CO_2 _at 37°C. When *in situ *run-on transcription was assessed, early one-cell embryos were treated with 100 nM flavopiridol or 250 μg/mL α-amanitin, or left untreated. Embryos were cultured for 9 h or 32 h and subjected to BrUTP labeling.

### Antibodies and reagents

A rabbit polyclonal antibody against CDK9 (Santa Cruz, sc-484) and a mouse monoclonal antibody against cyclin T1 (Santa Cruz, cs-271575) were diluted 1:50. Monoclonal antibodies against Pol II CTD phospho S2 (H5), Pol II CTD phospho S5 (H14), and hypophosphorylated Pol II CTD (8WG16), were purchased from Covance and diluted 1:50. To confirm the specificities of the anti-CDK9 and -cyclin T1 antibodies, working dilutions of the antibodies were added to the relevant antigen peptides at a molar ratio of 1:10. The antibody/peptide mixtures were next incubated with gentle shaking for 2 h at room temperature prior to use as immunostaining controls. Secondary antibodies were conjugated with FITC or Texas Red. Flavopiridol (F3055) and α-amanitin (A2263) were purchased from Sigma and dissolved in sterile double-distilled water to form 0.5 mM and 1 mg/mL stock solutions, respectively.

### Immunofluorescence staining

Oocytes and embryos were washed twice in 0.1% (w/v) polyvinyl alcohol in PBS (PBS-PVA) and fixed in 2% (v/v) formaldehyde in PBS for 15 minutes at room temperature. Next, oocytes were permeabilized for 15 minutes in 0.5% (v/v) Triton X-100 in PBS, washed for 10 minutes in 100 mM glycine in PBS (to inactivate free aldehyde groups), and nonspecific binding sites were blocked with 4% (w/v) bovine serum albumin for 10 minutes, followed by 5 minutes in PBG [PBS containing 0.5% (w/v) BSA and 0.1% (w/v) gelatin from the skin of cold-water fish (Sigma)]. Incubations with primary antibodies proceeded in PBG for 16 hours at 4°C. Cells were subsequently washed four times, for 5 minutes each time, in PBG, and incubated with the appropriate secondary antibodies for 1.5 hours in PBG at room temperature. Next, the cells were washed twice, for 5 minutes each time, in PBG and twice for 5 minutes each time in PBS. Chromatin was counterstained with DAPI for 10 min at RT. For microscopic observation, embryos were deposited on slides and mounted under coverslips using Vectashield (Vector Laboratories) mounting medium.

### *In situ *run-on transcription

*In situ *run-on transcription was performed as described earlier [42], with some modifications. Briefly, embryos were collected and rinsed with PBS, followed by incubation in physiological buffer (PB; 100 mM potassium acetate, 30 mM KCl, 10 mM Na_2_HPO_4_, 1 mM MgCl_2_, 1 mM Na_2_ATP, 1 mM DTT, and 0.2 mM PMSF; pH 7.2) with 100 μg/ml BSA and 80 U/mL RNasin. Embryos were incubated on ice and permeabilized with PB containing 0.05% (v/v) Triton X-100 for 2 min at room temperature, followed by washing with PB and further incubation with transcription mix (PB supplemented with 100 μM ATP, CTP, GTP, and Br-UTP; and 1 mM MgCl_2_). Labeling was performed for 15 min at 33°C. Embryos were subsequently washed with PB and repermeabilized with PB containing 0.2% (v/v) Triton X-100 for 2 min at 4°C, followed by fixation with 2.5% (w/v) PFA for 20 min at room temperature and blocking with 2% (w/v) BSA in PBS. Immunolabeling was achieved by overnight incubation with mouse monoclonal anti-BrdU antibody (Sigma B8434), followed by washing and further incubation for 1 h with FITC-conjugated mouse IgG. Chromatin was counterstained with DAPI.

### Confocal microscopy and fluorescence intensity measurement

Images were captured using a Zeiss scanning laser confocal microscope running Zeiss LSM Image Browser software. Serial optical sections (the Z-series) were collected at 1 μm intervals; all nuclear and cytoplasmic regions were covered. The Z-series were stacked and images depicting staining patterns and intensities of all nuclear and cytoplasmic entities were generated. All samples of oocytes and embryos were prepared and processed simultaneously prior to fluorescence intensity measurements. The laser power was adjusted to ensure that signal intensity was below saturation for the developmental stage that displayed the highest intensity and all images were next scanned at that laser power. All images in any particular developmental series were acquired using the same laser power output. To quantify fluorescence intensity, nuclear signals were outlined and mean fluorescence intensity measured. Such encircled regions were software-dragged into the cytoplasm of the same cell, and background fluorescence was next measured. Each specific signal was calculated by dividing the nuclear value by the cytoplasmic value.

## Authors' contributions

RKO performed most of the experiments and wrote the paper. HRK and YFD prepared and cultured mouse embryos. CSP and DIJ designed the study and revised the paper. All authors read and approved of the final version of the manuscript.
